# Sexual happiness and satisfaction with sexual safety among German trans men who have sex with men: results from EMIS‐2017

**DOI:** 10.1002/jia2.25992

**Published:** 2022-10-12

**Authors:** Max Nicolai Appenroth, Uwe Koppe, Ford Hickson, Susanne Schink, Alexander Hahne, Axel J. Schmidt, Peter Weatherburn, Ulrich Marcus

**Affiliations:** ^1^ Department of Infectious Disease Epidemiology Robert Koch‐Institute Berlin Germany; ^2^ Institute of Public Health Charité Universitätsmedizin Berlin Berlin Germany; ^3^ Department of Public Health Environments & Society Faculty of Public Health & Policy London School of Hygiene & Tropical Medicine London UK; ^4^ Freelance Sex Educator and Bodyworker Hamburg Germany

**Keywords:** trans MSM, trans men, gender diversity, HIV prevention, sexual happiness, MSM

## Abstract

**Introduction:**

The population of men‐who‐have‐sex‐with‐men (MSM) includes people who are on the masculine spectrum but were assigned female at birth (AFAB), that is trans MSM. This study aims to identify current circumstances regarding sexual happiness and safety among German trans MSM. To date, there is no health information about trans MSM in Germany, limiting the ability of MSM sexual health programmes to meet their needs.

**Methods:**

Data were used from the European MSM Internet Survey (EMIS‐2017), where people identifying as men and/or trans men were recruited through dating apps for MSM, community websites and social media to participate in an online survey. We analysed parameters on sexual happiness and satisfaction with sexual safety among Germany‐based trans MSM and compared those to outcomes of MSM assigned male at birth (cis MSM) living in Germany using descriptive methods and logistic regression models adjusting for age.

**Results:**

In total, 23,001 participants from Germany were included, of which 122 (0.5%) indicated to be AFAB (i.e. trans MSM). Trans MSM were markedly younger than cis participants (median age: 28.5 vs. 39 years).

Trans MSM more often reported being unhappy with their current sex life (adjusted odds ratio [aOR] = 1.82, 95% CI 1.24–2.67), had higher odds of disagreeing with the statements “the sex I have is always as safe as I want” ([aOR] = 1.82, 95% CI 1.24–2.67) and “I find it easy to say no to sex that I don't want” ([aOR] = 1.80, 95% CI 1.18–2.77).

Trans MSM were more likely to not be living comfortably financially ([aOR] = 2.43, 95% CI 1.60–3.67) and to be living with severe anxiety and/or depression ([aOR] = 3.90, 95% CI 2.22–6.83). Trans MSM were less likely to have ever tested for HIV ([aOR] = 0.63, 95% CI 0.43–0.93).

**Conclusions:**

Sexual happiness, control of sexual boundaries, satisfaction with sexual safety, financial security, mental wellbeing and HIV testing were all lower in German trans MSM compared with cis MSM. Tailored sexual health interventions, contextualized with regard to needs and vulnerabilities, could address this inequality.

## 1 INTRODUCTION

In the past 5 years, human immunodeficiency virus (HIV) prevention and research among men‐who‐have‐sex‐with‐men (MSM) has increasingly included transmasculine people. Yet, little is known about individuals who are on the masculine spectrum and whose gender is different from their sex assigned at birth. HIV prevention and other sexual health data about trans MSM are scarce.

The World Health Organization has declared trans people as a key population in regard to HIV exposure [1] and this community, especially trans women of colour, is disproportionately affected by HIV and other sexually transmitted infections (STIs) [2, 3, 4]. However, little is known about trans community members who were assigned female at birth (AFAB; i.e. trans men, transmasculine individuals and AFAB men) [2]. A U.S.‐based analysis of trans‐inclusive research found laboratory‐confirmed HIV infections in 3.2% of transmasculine participants [3]. Estimations suggest that currently, 1.2 million people are living with HIV in the United States [5], which represents about 0.36% of the U.S. population. Accordingly, transmasculine people appear to be more likely living with HIV than the general population. Due to a lack of research, estimations about HIV prevalence in transmasculine communities in other global regions are not possible at this point.

Although experiencing a possible elevated risk for HIV, testing rates among transmasculine people appear lower compared to cis gay and bisexual men [6]. Additionally, access and uptake of HIV pre‐exposure prophylaxis (PrEP) have been limited in this group [7, 8]. Both issues were associated with poor knowledge of healthcare providers about the specific HIV risks and vulnerabilities of transmasculine individuals [6]. Many transmasculine people engage sexually with cis men [9, 10, 11, 12]. Physical changes accompanying gender‐affirming hormones (i.e. vaginal/front hole tissue changes and a greater need for using lubricants, when engaging in vaginal/front hole sex) [13] and difficulties navigating safer sex discussions [14, 15, 16] put transmasculine people at risk for STI/HIV infections. This risk comes alongside a lack of knowledge about trans‐lived realities among healthcare providers. In healthcare settings, trans people are often confronted with gendered body stereotypes (e.g. norms like “all men have a penis”), heteronormative expectations (e.g. “trans men sexually engage only with cis women”), lack of trans‐competent treatment knowledge by healthcare providers [17], alongside experiences of discrimination [18, 19, 20, 21].

Overall sexual satisfaction in trans communities is understudied. Barriers to sexual satisfaction among trans individuals are difficulties creating sexual encounters and being afraid of sexual contact in general [22]. A study sample (*n* = 518) collected at three gender clinics in Belgium, the Netherlands and Germany included results of 307 trans women and 211 trans men. The results showed that 26% of trans women and 32% of trans men who indicated sexual problems found it difficult to initiate sexual contact. Additionally, 21% of trans women and 22% of trans men reported being afraid of sexual contact. Another study with cis and trans participants from Canada and the United States, regardless of their sexual orientation, found that 87.5% would not date a trans person [23]. Heterosexual cis men (96.7%) and cis women (98.2%) were most likely not to be interested in dating a trans person. Respondents identifying as bisexual, queer or non‐binary (48.3%) were more likely to consider trans individuals as potential dating partners. An Australian‐based study among trans people showed that 42.2% of trans men were anxious when thinking about their sex life [24].

When discussing a fulfilling sexual life in trans communities, it is crucial to acknowledge the importance of gender affirmation (e.g. being gendered correctly by others). Gender affirmation is directly linked to improved mental health [25], and although not all trans people undergo physical changes to align their bodies with their gender identity, access to such gender‐affirming treatment minimizes negative body images [26]. Gender‐confirming treatment has a positive influence on sexual feeling in trans women, but a greater impact is attributed to bodily satisfaction (e.g. feeling comfortable in a person's own body) [27]. Sexual confidence significantly improved in AFAB trans people who underwent masculinizing chest surgery [27, 28]. Besides multiple barriers to healthcare, the research found that sexual body image worries in trans populations are linked with poor sexual health outcomes. Higher self‐esteem and sexual satisfaction were associated with stronger condom negotiation skills [29].

Currently, information on sexual happiness and sexual safety among trans MSM in Germany is lacking. The data about this group in Germany, collected through the European MSM Internet Survey 2017 (EMIS‐2017) and presented in this article, is the first of its kind, and it will depict risks and vulnerabilities in regard to HIV/STIs faced by this community.

## 2 METHODS

The data used for this analysis come from the European MSM Internet Survey 2017, a community‐recruited online survey (EMIS‐2017; www.emis2017.eu). Fieldwork occurred from 9 October 2017 to 31 January 2018 for self‐completion of the questionnaire. Community‐based recruitment occurred on targeted websites, apps and social media. Responses were included if individuals: identified as MSM, were legally old enough (in their country) to have sex with men, understood the purpose of the study and gave their consent to participate. A more detailed description of the methods has been published previously [30].

Based on the German EMIS sub‐sample, we compared demographics, sexual behaviour, sexual happiness and satisfaction with sexual safety among German trans MSM with outcomes of German cis MSM using descriptive methods and logistic regression models adjusting for age.

### 2.1 Participants

The analytic sub‐sample for this paper was EMIS respondents living in Germany who provided valid responses about their sex assigned at birth and current gender identity.

In this report, we define trans MSM as people who are “men” or “trans men” (by self‐identification) and female assigned at birth, and who are sexually attracted to and/or have sex with men. “Men” assigned male at birth (AMAB) are referred to as “cis” in this analysis. “Trans men” AMAB were excluded from this study.

### 2.2 Outcome variables

The study asked for a number of demographic and sexual health information. The way in which questions were asked in detail with answer options has been described elsewhere [14].

Age was recorded in years and collapsed into five categories (14–17; 18–29; 30–39; 40–49; 50 and older). Financial coping was categorized into “living comfortably” and “not living comfortably” on current income. The sexual identity included the answers “gay/homosexual,” “straight/heterosexual,” “bisexual,” “any other term” and “I don't usually use a term.” Partnership status was dichotomized as “single or unsure” and “steady partner,” and HIV diagnosis was captured through a “yes” or “no” answer to the question of whether participants ever received a positive HIV test result.

As for mental health, EMIS‐2017 used the PHQ‐4 to provide a combined indicator for anxiety and depression. Answers were measured with a standardized system of “normal,” “mild,” “moderate” and “severe.” The question about feeling suicidal was categorized into “yes, at least some days” or “never.”

A numerical scale from 1 to 10 was offered to gather data about sexual happiness, and participants were asked “On a scale of 1 to 10 (where 1 is the most unhappy and 10 is the most happy), how happy are you with your sex life?” Answers were dichotomized into “unhappy” (1–4) and “happy” (5–10).

The answer regarding the number of steady male sexual partners in the past 12 months was categorized as “0,” “1,” “2,” or “3 or more,” and the answer to the question about the number of non‐steady male sexual partners in the past 12 months was grouped into four categories (0, 1–3, 4–10 and 11 or more).

Participants were asked whether they “agree” or “disagree” with the statements “the sex I have is always as safe as I want to be” and “I find it easy to say ‘no’ to sex I don't want” to assess their safer sex self‐efficacy [14].

Questions regarding HIV testing and prevention (ever having received an HIV test result; ever had an STI test other than HIV; ever heard of PrEP; ever used PrEP; ever talked to a healthcare provider about PrEP) were dichotomized to “yes” or “no.”

### 2.3 Ethical approval

The study received approval from the Observational & Interventions Research Ethics Committee of the London School of Hygiene & Tropical Medicine (14 September 2017; LSHTM ethics ref: 14421) [14].

## 3 RESULTS

We included 23,001 individuals living in Germany participating in EMIS‐2017 who either reported that they were AMAB and identified as men (*n* = 22,879; 99.5%) or who indicated having been AFAB but who identified as trans men (*n* = 95; 0.4%) or men (*n* = 27; 0.1%). AFAB trans men and men were grouped into the category of trans MSM (*n* = 122) for this analysis. The 56 respondents who indicated being AMAB and who identified as a “trans man” were excluded from this analysis.

### 3.1 Demographics

Participating trans MSM were considerably younger (median age 28.5 years [IQR 23–37]) compared to cis MSM (39 years [IQR 29–49]). Over half (52.5%) of trans MSM were aged 18–29 compared to about a quarter (26.4%) of cis MSM.

Trans MSM were much more likely to not be living comfortably on their current income (74.6% vs. 49.9%, age‐adjusted odds ratio [aOR] = 2.43, 95% CI = 1.60–3.67) compared to cis MSM (Table 1).

**Table 1:** Demographic and mental health data of trans MSM and cis MSM EMIS participants (*N* = 23,001)

Variable	Trans MSM	Cis MSM	Univariable regression^a^	Regression adjusted for age^b^	*p*‐value^c^
Overall	122 (0.5%)	22,879 (99.5%)	–	–	
Age (years)					
Median (IQR)	28.5 (23–37)	39 (29–49)	–	–	
14–17	4 (3.3%)	197 (0.9%)	1.92 (0.69–5.32)	–	0.211
18–29	64 (52.5%)	6043 (26.4%)	1	–	–
30–39	27 (22.1%)	5681 (24.8%)	0.45 (0.29–0.70)	–	**0.001**
40–49	19 (15.6%)	5382 (23.5%)	0.33 (0.20–0.56)	–	**<0.001**
50 and older	8 (6.5%)	5576 (24.4%)	0.14 (0.06–0.28)	–	**<0.001**
Income					
Living comfortably	31 (25.4%)	11,466 (50.1%)	1	1	
Not living comfortably	91 (74.6%)	11,413 (49.9%)	2.98 (1.98–4.48)	2.43 (1.60–3.67)	**<0.001**
Sexual identity					
Gay or homosexual	59 (48.4%)	17,918 (78.3%)	0.60 (0.36–0.99)	0.56 (0.34–0.93)	**0.024**
Bisexual	21 (17.2%)	3818 (16.7%)	1	1	
Straight or heterosexual	2 (1.6%)	125 (0.6%)	2.91 (0.67–12.54)	2.63 (0.61–11.37)	0.196
Any other term	23 (18.9%)	175 (0.8%)	23.89 (12.97–44.01)	16.49 (8.87–30.66)	**<0.001**
I don't usually use a term	17 (13.9%)	824 (3.6%)	3.75 (1.97–7.14)	3.24 (1.70–6.20)	**<0.001**
Missing	–	19 (0.1%)	–	–	
Partnership status					
Single or unsure	80 (65.6%)	12,257 (53.6%)	1.65 (1.13–2.40)	1.23 (0.84–1.81)	0.284
Steady partner	42 (34.4%)	10,599 (46.3%)	1	1	
Living with depression/anxiety					
Normal	42 (34.4%)	13,463 (58.8%)	1	1	
Mild	44 (36.1%)	6190 (27.1%)	2.28 (1.49–3.48)	1.97 (1.29–3.02)	**0.002**
Moderate	15 (12.3%)	1729 (7.6%)	2.78 (1.54–5.03)	2.14 (1.18–3.88)	**0.012**
Severe	18 (14.8%)	1133 (5%)	5.09 (2.92–8.88)	3.90 (2.22–6.83)	**<0.001**
Missing	3 (2.5)	364 (1.6%)			
Suicidal ideation					
Yes, at least some days	50 (41%)	3523 (15.5%)	3.79 (2.64–5.44)	3.27 (2.27–4.72)	**<0.001**
Never	72 (59%)	19,211 (84.5%)	1	1	
Missing	–	–			

^a^ Univariable logistic regression model with 122 trans and 22,879 cis EMIS‐2017 participants.

^b^ Multivariable logistic regression model with 122 trans and 22,879 cis EMIS‐2017 participants adjusting for age.

^c^ *p*‐values of age‐adjusted regression. Statistically significant *p*‐values (*p* < 0.05) are shown in bold.

Abbreviations: EMIS, European MSM Internet Survey; IQR, interquartile range; MSM, men who have sex with men.

### 3.2 Sexuality and relationship status

While similar proportions of trans and cis MSM identified as “bisexual” (17.2% and 16.7%), trans MSM were less likely to identify as “gay” or “homosexual” (48.4% vs. 78.3%; [aOR] = 0.56, 95% CI = 0.34–0.93) and were more likely to use other terms (18.9% vs. 0.8%; [aOR] = 16.49, 95% CI = 8.87–30.66) or no term (13.9% vs. 3.6%; [aOR] = 3.24, 95% CI = 1.70–6.20). Trans MSM (65.6%) were numerically more likely to report being single or of unsure relationship status (vs. 53.6% in cis MSM; [aOR] = 1.23, 95% CI = 0.84–1.81) (Table 1).

### 3.3 Mental health

14.8% of trans MSM had a PHQ‐4 score suggesting they are living with depression and/or anxiety compared to 5.0% of cis MSM ([aOR] = 3.90, 95% CI 2.22–6.83).

Trans MSM were more likely than cis respondents to feeling suicidal on some days ([aOR] = 3.27, 95% CI 2.27–4.72) (Table 1).

### 3.4 Sexual happiness and satisfaction with sexual safety

Trans MSM were more likely than cis MSM to report being unhappy with their current sexual life (33.6% vs. 22.3%; [aOR] = 1.82, 95% CI 1.24–2.67). Additionally, they were more likely to disagree with the statements “The sex I have is always as safe as I want to be” ([aOR] = 1.77, 95% CI 1.11–2.82) and “I find it easy to say ‘no’ to sex I don't want” ([aOR] = 1.80, 95% CI 1.18–2.77) (Table 2).

**Table 2:** Sexual behaviour and HIV/STI prevention data of trans MSM and cis MSM EMIS‐2017 participants (*N* = 23,001)

	Trans MSM	Cis MSM	Univariable regression^a^	Regression adjusted for age^b^	*p*‐value^c^
Overall	122 (0.5%)	22,879 (99.5%)			
Sexual happiness					
Unhappy (1–4)	41 (33.6%)	5106 (22.3%)	1.89 (1.29–2.77)	1.82 (1.24–2.67)	0.002
Happy (5–10)	73 (59.8%)	17,182 (75.1%)	1	1	–
Missing	8 (6.6%)	591 (2.6%)	–	–	–
Sex is always as safe as I want					
Agree	100 (82%)	20,386 (89.1%)	1	1	–
Disagree	22 (18%)	2394 (10.5%)	1.87 (1.18–2.98)	1.77 (1.11–2.82)	**0.016**
Missing	–	99 (0.4%)	–	–	–
I find it easy to say no to sex I don't want					
Agree	94 (77.1%)	19,952 (87.2%)	1	1	–
Disagree	28 (23%)	2767 (12.1%)	2.15 (1.41–3.28)	1.80 (1.18–2.77)	**<0.001**
Missing	–	160 (0.7%)	–	–	–
Number of steady sexual partners in the past 12 months					
0	92 (75.4%)	13,350 (58.4%)	1	1	–
1	27 (22.1%)	6940 (30.3%)	0.56 (0.37–0.87)	0.51 (0.33–0.79)	**0.002**
2	3 (2.5%)	1074 (4.6%)	0.42 (0.31–1.32)	0.40 (0.13–1.28)	0.123
3 or more	0 (0%)	1348 (5.9%)	–	–	–
Missing	–	194 (0.9%)	–	–	–
Number of non‐steady sexual partners in the past 12 months					
0	74 (60.7%)	8531 (37.3%)	1	1	–
1–3	28 (22%)	6129 (26.8%)	0.53 (0.34–0.81)	0.54 (0.35–0.84)	**0.006**
4–10	13 (10.7%)	4373 (19.1%)	0.34 (0.19–0.62)	0.36 (0.20–0.64)	**0.001**
11 or more	7 (5.7%)	3518 (15.4%)	0.23 (0.11–0.50)	0.26 (0.12–0.57)	**0.001**
Missing	–	328 (1.4)	–	–	
Chemsex in the past 12 months					
Yes	10 (8.2%)	2145 (9.4%)	0.86 (0.45–1.64)	0.89 (0.46–1.70)	0.715
No	111 (91%)	20,420 (89.3%)	1	1	–
Missing	1 (0.8%)	314 (1.4%)	–	–	–
Received HIV‐positive diagnosis					
Yes	3 (2.5%)	2448 (10.7%)	0.21 (0.07–0.66)	0.33 (0.10–1.04)	0.059
No	118 (96.7%)	20,242 (88.5%)	1	1	–
Missing	1 (0.8%)	189 (0.8%)	–	–	–
Ever received an HIV test result					
Yes	71 (58.2%)	17,411 (76.1%)	0.44 (0.31–0.63)	0.63 (0.43–0.93)	**0.018**
No	50 (41%)	5390 (23.6%)	1	1	–
Missing	1 (0.8%)	78 (0.3%)	–	–	–
Ever tested for STIs					
Yes	55 (45.1%)	12,427 (54.3%)	0.67 (0.47–0.96)	0.84 (0.58–1.21)	0.358
No	67 (54.9%)	10,215 (44.7%)	1	1	–
Missing	–	237 (1%)	–	–	–
Ever heard of PrEP					
Yes	55 (45.1%)	13,567 (59.3%)	0.78 (0.55–1.12)	0.81 (0.57–1.16)	0.256
No	66 (54.1%)	8872 (38.8%)	1	1	–
Missing	1 (0.8%)	440 (1.9%)	–	–	–
Ever talked to healthcare provider about PrEP					
Yes	2 (1.6%)	1644 (7.2%)	0.22 (0.05–0.87)	0.22 (0.06–0.91)	**0.036**
No	119 (97.5%)	21,121 (92.3%)	1	1	–
Missing	1 (0.8%)	114 (0.5%)	–	–	–
Ever used PrEP					
Yes	1 (0.8%)	491 (2.2%)	1	1	–
No	121 (99.2%)	22,234 (97.2%)	0.37 (0.05–2.68)	0.40 (0.06–2.88)	0.363
Missing	–	154 (0.7%)	–	–	–

^a^ Univariable logistic regression model with 122 trans and 22,879 cis EMIS‐2017 participants.

^b^ Multivariable logistic regression model with 122 trans and 22,879 cis EMIS‐2017 participants adjusting for age.

^c^ *p*‐values of adjusted regression. Statistically significant *p*‐values (*p* < 0.05) are shown in bold.

Abbreviations: EMIS‐2017, European MSM Internet Survey 2017; MSM, men who have sex with men; PrEP, pre‐exposure prophylaxis; STI, sexually transmitted infections.

### 3.5 Sexual behaviour

About three quarters (75.4%) of trans participants reported having no steady sexual partner (vs. 58.4% of cis MSM) and trans MSM were less likely to have multiple non‐steady sexual partners (1–3 non‐steady sexual partners [aOR] = 0.54, 95% CI 0.35–0.84; 4–10 non‐steady sexual partners [aOR] = 0.36, 95% CI 0.20–0.64; 11 or more non‐steady sexual partners [aOR] = 0.26, 95% CI 0.12–0.57) compared to the cis sample. Engagement in stimulant drug use for sex (chemsex) in the past 12 months prior to the study was comparable between the study groups ([aOR] = 0.89, 95% CI 0.46–1.70) (Table 2).

### 3.6 HIV and HIV prevention

Trans MSM were less likely to have ever received an HIV test result (58.2% vs. 76.1%; [aOR] = 0.63, 95% CI 0.43–0.93) and were less likely to have been diagnosed with HIV (2.5% vs. 10.7%; [aOR] = 0.33, 95% CI 0.10–1.04) compared to cis MSM.

Ever having been tested for (non‐HIV) STIs was less common than HIV testing among both study groups. Even though the proportion of trans MSM that tested for other STIs was numerically smaller than for cis MSM, the confidence interval overlaps the null value, and this difference might have arisen by chance (reported 45.1% vs. 54.3%; [aOR] = 0.84, 95% CI 0.58–1.21).

Trans MSM were numerically less likely to have heard of PrEP (45.1% vs. 59.3%; [aOR] = 0.81, 95% CI 0.57–1.16) and were also less likely to have talked to a healthcare provider about PrEP (1.6% vs. 7.2%; [aOR] = 0.22, 95% CI 0.06–0.91). Subsequently, while PrEP use was uncommon overall, it was numerically even less likely to have ever been used by trans MSM (0.8% vs. 2.8%; [aOR] = 0.40, 95% CI 0.06–2.88) (Table 2).

## 4 DISCUSSION

The data analysis from the European MSM Internet Survey 2017 (EMIS‐2017) demonstrates differences in a range of sexual health indicators between trans and cis MSM in Germany. Trans MSM were less likely to access sexual health services (spoken to about PrEP and received HIV/STI test results) and were less likely to have their sexual health needs met (being aware of PrEP, being able to say “no” and only doing things I don't regret). They were also less likely to engage in sexual risk behaviours (multiple partners) and less likely to engage in precautionary behaviour (taking PrEP). They were both less likely to be living with diagnosed HIV and less likely to be happy with their sex life.

These differences were large enough to be detected despite a relatively small number of trans MSM in the sample. The findings present a first outline of the sexual health profile of trans MSM in Germany.

Looking at the results of this study, trans MSM were more likely to not live comfortable financially. Socio‐economic disadvantages in trans MSM found here align with previous findings [18, 31, 32]. This may be attributed to the relatively younger age of trans participants, but also to discriminatory experiences in education and work settings [18, 21].

The high levels of mental health problems and suicidality among trans MSM participants of the EMIS‐2017 align with previous research showing that trans individuals are disproportionately affected by mental health‐related issues and suicidal ideation [18]. Although studies have found higher HIV testing rates among people living with mental health problems, HIV prevalence is higher among people affected by poor mental health [33]. It is unclear if higher testing rates can be found among transmasculine individuals living with mental health‐related problems, as testing rates are comparably low in this group [6]. Trans MSM could specifically benefit from combined and integrated mental and sexual health services.

Sexual risk behaviour measured by the number of sexual partners differed within both study groups. Cis participants were more likely to engage more with steady and non‐steady sexual partners compared to trans participants. This finding may reflect results from other studies where trans participants reported difficulties initiating sex and fear of sexual activity [22, 24]. Barriers of finding sexual contacts and that trans people are not considered as dating partners may account for lower numbers of sexual partners found in this analysis.

Additionally, sexual unhappiness in trans MSM may be directly linked to difficulties in finding sexual partners. This study showed that cis participants were more likely to be satisfied with their current sexual life, and more trans MSM indicated not being satisfied with their sex life. Sexual (dis‐) satisfaction in trans MSM may be directly linked to gender dis‐affirmation by cis MSM, leading to higher levels of psychological distress and anxiety in trans MSM [34].

This data analysis suggests lower levels of HIV testing among trans MSM and even lower frequencies for other STI testing. This finding aligns with other research reporting low testing frequencies in trans populations [6, 35]. A cross‐sectional online study in the United States found high rates of trans MSM who have never tested for HIV or bacterial and viral STIs, especially among younger participants [36]. Trans MSM receiving positive gender affirmation by cis MSM had higher HIV testing frequency [34].

Lower testing rates might be associated with negative experiences of trans people in healthcare settings. An analysis based on the 2015 U.S. Transgender Survey showed that specifically transmasculine participants postponed or even avoided seeking healthcare due to anticipated discrimination in healthcare settings [32]. A European‐wide study among Lesbian, Gay, Bisexual, Trans and Inter people found that 34% of the trans respondents experienced discrimination in healthcare or social service settings, with disproportionately higher rates in Germany (40%) [21]. A previous study among trans people in Europe showed that trans men were especially vulnerable to discrimination by healthcare providers [37]. Besides the experiences of discrimination, stereotypical assumptions about the sexuality and sexual practices of trans MSM may lead to inadequate service provision [38]. A poor risk assessment by healthcare providers, specifically in the field of sexual health, may cause a lack of appropriate testing and prevention opportunities.

The analysed data were collected in 2017 before the formal rollout of PrEP in Germany. Lacking knowledge on the side of healthcare providers about lived realities of trans MSM may contribute to the fact that trans MSM in this sample were less likely to have heard about PrEP, talked to a healthcare provider about PrEP or ever used PrEP. These findings align with previous studies that showed low PrEP uptake in transmasculine individuals and a lack of conversations with healthcare providers about this drug [7] and country‐specific barriers to PrEP uptake in trans individuals in Germany have been described previously [39]. That 2.5% of the trans MSM in this sample are living with diagnosed HIV illustrates the large benefit gap when only 0.8% of those who are not positive are using PrEP. While trans MSM group risk may be lower than that of cis MSM, it is higher than that of the general population (see Introduction). PrEP services and promotions for MSM should be trans inclusive, and trans MSM‐specific programmes should be considered.

In 2020, the Deutsche Aidshilfe (German AIDS Service Organization) published a brochure developed by and targeted to transmasculine individuals who have sex with men [40]. This brochure is the only published sexual health information inclusive of the target population in German. Regarding service provision, for example, CliniQ in London/UK is a sexual health clinic operated by and for trans people (https://cliniq.org.uk/). Given the shared experience of discrimination in healthcare settings by trans people and the reduced risk for HIV acquisition through peer‐led education [41], peer‐led sexual health services are a very much‐needed intervention for the trans community. However, such services are only offered periodically in two cities in Germany. The Checkpoint BLN (Berlin) offers peer‐testing and counselling for trans, non‐binary and inter individuals once a month [42]. The Münchner Aids‐Hilfe (Munich AIDS Service Organization) runs a counselling service for trans and inter people. Every 3 months for 3 hours, HIV/STI‐testing is offered on a peer basis [43]. Both opportunities are for the wider trans and inter community, and sexual health services specifically targeted at transmasculine identities are missing.

The study has a few limitations. The small sample size of German trans MSM in this study only allows a small insight into the lived experiences of this community, and further research is needed. MSM recruited online differ from the general MSM population by over‐representing MSM identifying as gay and reporting more sexual risk behaviours [44]. In all self‐selection surveys, participants with lower education levels are underrepresented.

We are aware that grouping together “trans men” and people AFAB who refer to themselves simply as “men” is not ideal, as the latter group might reject an identification as trans. However, for more appropriate analysis, this seemed like the best choice, but we wish to highlight that the matter of self‐identification of trans people is a sensitive topic.

However, this analysis opens a path for a better understanding of the needs of trans MSM and the possibility to target their sexual health needs in a more appropriate way. Trans community members were consulted to review measures on gender identity and sex assigned at birth, and the analysis, as well as drafting of the manuscript, have been conducted by a transmasculine researcher from Germany.

## 5 CONCLUSIONS

This research presents the first data about trans men and AFAB men who have sex with men (trans MSM) living in Germany and shows their comparative disadvantage. The outcomes demonstrate complex aspects of sexual happiness of trans MSM, negotiating safer sex and sexual boundaries. Lower uptake of HIV and STI testing and talking to healthcare providers about HIV prevention methods, such as PrEP, may be connected to potential experiences of discrimination in healthcare settings faced by many trans people.

Sexual health services need to expand their efforts to include this population in their prevention strategies, outreach and care. For example, community‐informed safer sex negotiation and sexual boundary trainings or peer‐led sexual health interventions may reduce the overall risk of HIV/STI exposure, improve the uptake of sexual health services and enhance satisfaction with sexual life in trans MSM. Taking these outcomes and other existing data into account, sexual health interventions need to be tailored to meet the needs and vulnerabilities of trans MSM in the German context and beyond.

## COMPETING INTERESTS

The authors declare no competing interests.

The funder defined the primary population (men who have sex with men) and morbidities (sexually transmitted infections) of concern. The funder had no role in the collection, analyses or interpretation of data, in the writing of the manuscript, or in the decision to publish the results.

## AUTHORS’ CONTRIBUTIONS

AJS, FH, PW and UM designed the study. SS and UM prepared the German sub‐data set for pre‐analysis. MNA, UK and UM performed the analysis presented in this manuscript. MNA wrote the first original draft. All authors reviewed and edited the manuscript.

## FUNDING

This research was funded by the European Union in the framework of the Third EU Health Programme (2014–2020).

Data requests should be addressed to the London School of Hygiene and Tropical Medicine Research Operations Office Data Management Lead (alex.hollander@lshtm.ac.uk), the first author (Max.appenroth@charite.de) and the Principal Investigator of EMIS‐2017 (Peter.Weatherburn@lshtm.ac.uk). Individuals requesting data should present their research objective(s) and enclose a list of requested variables. To protect the confidentiality of participants, data sharing is contingent upon appropriate data handling and good scientific practice by the person requesting the data and should furthermore be in accordance with all applicable local requirements.

The London School of Hygiene and Tropical Medicine administrative offices are located at Keppel Street, London WC1E 7HT, United Kingdom.

## Data Availability

The EMIS‐2017 dataset used for this analysis has been obtained from the London School of Hygiene and Tropical Medicine under a data transfer agreement that prohibits sharing the dataset publicly. Although we cannot make study data publicly accessible at the time of publication, all authors commit to make the data underlying the findings of the study available in compliance with the JIAS Data Availability Policy.
